# Cardiovascular diseases and risk factors associated with sudden cardiac death in amateur athletes: a scoping review

**DOI:** 10.3389/fpubh.2026.1770168

**Published:** 2026-02-03

**Authors:** Leonardo Arzayus-Patiño, Yiseth F. Carabali-Bonilla, María C. Mora-Salazar, Carolina Castro Gómez, Vicente Benavides-Cordoba

**Affiliations:** 1 Programa de Fisioterapia, Facultad de Salud, Universidad Santiago de Cali, Cali, Colombia; 2 Institución Universitaria Escuela Nacional del Deporte, Cali, Colombia; 3 Fundación Valle del Lili, Medicina Física y Rehabilitación, área de Rehabilitación Cardiaca, Cali, Colombia; 4 Facultad de Salud, Universidad del Valle, Cali, Colombia

**Keywords:** amateur athletes, cardiovascular diseases, risk factors, sudden cardiac arrest, sudden cardiac death

## Abstract

**Background:**

Sudden cardiac death (SCD) is a rare but devastating event in the sports setting, often affecting apparently healthy and physically active individuals. Although regular physical activity is widely promoted as a protective factor against cardiovascular disease, cases of SCD continue to be reported not only in elite athletes but also in amateur and recreational athletes, who frequently lack systematic cardiovascular screening.

**Objective:**

To map the available evidence on the most prevalent cardiovascular diseases and the associated risk factors related to sudden cardiac death in amateur athletes.

**Methods:**

A scoping review was conducted following the PRISMA-ScR guidelines and the methodological framework proposed by the Joanna Briggs Institute (JBI). The research question was structured using the PCC framework (Population: amateur athletes aged ≥18 years; Concept: cardiovascular diseases and associated risk factors; Context: sudden cardiac death). Systematic searches were performed in PubMed, Scopus, SciELO, and Springer, with no restrictions on publication date and including studies published in English, Spanish, and Portuguese. Study selection, data extraction, and methodological quality assessment were independently performed by two reviewers, with disagreements resolved by a third reviewer. Methodological quality was assessed using JBI critical appraisal tools for observational cohort studies.

**Results:**

A total of 1,807 records were identified, of which five observational studies met the inclusion criteria. The most frequently reported cause of SCD in amateur athletes was hypertrophic cardiomyopathy, followed by atherosclerotic coronary artery disease—particularly in athletes older than 35 years—and myocarditis, mainly in younger individuals with recent respiratory infections. Football was the sport most commonly associated with SCD events. The main risk factors identified included male sex, intense physical exertion, traditional cardiovascular risk factors (smoking, hypertension, dyslipidemia, prior myocardial infarction, and coronary stenosis), family history of premature coronary disease, and the absence of early cardiopulmonary resuscitation or defibrillation at the event site. Overall methodological quality ranged from moderate to high.

**Conclusion:**

Sudden cardiac death in amateur athletes is predominantly associated with underlying cardiovascular diseases, particularly hypertrophic cardiomyopathy, and with a combination of modifiable and non-modifiable risk factors. These findings highlight that SCD is not exclusive to elite sports and underscore the need for preventive strategies in amateur athletes, including cardiovascular screening, risk factor control, education in cardiopulmonary resuscitation, and availability of automated external defibrillators in sports settings.

## Introduction

Sudden cardiac death (SCD) represents a clinically significant event in the sports setting, as it may occur in individuals who appear healthy and are physically active. Sudden cardiac death is commonly defined as death occurring unexpectedly within the first hour after the onset of symptoms (25). Although its incidence is relatively low, its unpredictable and often fatal nature generates increasing concern. This phenomenon has attracted considerable interest from the medical and scientific community, particularly because it affects both professional and amateur athletes in a context where physical activity is widely promoted as a strategy to prevent chronic diseases, including cardiovascular conditions (1).

Several studies have shown that the incidence and causes of SCD vary according to the type of sport, the athlete’s age, and the level of competition (2). In young athletes, the primary causes are mainly related to inherited structural heart diseases and channelopathies, whereas in individuals older than 35 years, atherosclerotic coronary artery disease is more prevalent. In countries such as the United States and several European nations, sports such as soccer and basketball present the highest incidence rates, highlighting the need to develop sport- and population-specific preventive strategies (3).

The situation of amateur athletes is particularly concerning, as unlike professional athletes, they are less likely to undergo comprehensive medical evaluations. The absence of pre-participation cardiovascular screening, such as electrocardiography and echocardiography, limits the early detection of conditions such as hypertrophic cardiomyopathy, hypertension, or coronary artery disease, all of which are closely associated with SCD. In addition, modifiable risk factors such as the use of stimulant substances, smoking, and obesity are common among physically active individuals, revealing a contradiction between sports participation and true cardiovascular protection. This underscores the importance of incorporating cardiovascular health as a central component of training programs, regardless of the competitive level (4).

Although regular physical activity is widely recognized as essential for controlling cardiovascular risk factors, the continued occurrence of SCD among apparently healthy amateur athletes highlights an important gap in current knowledge (5). Many of these individuals lack evident clinical histories or abnormalities in conventional risk factors, raising the question of whether other, less investigated or undiagnosed factors may increase the likelihood of fatal events even in physically active populations.

In this context, it is relevant to systematically analyze which cardiovascular diseases and risk factors are associated with SCD in amateur athletes, as well as to explore their relationship with different sporting practices. Such an approach may help guide more effective strategies for early detection, clinical monitoring, and individualized prevention in this population (6, 7). Therefore, the following research question arises:

What are the most prevalent cardiovascular diseases and risk factors associated with sudden cardiac death in amateur athletes? A scoping review.

## Materials and methods

This scoping review was conducted following the guidelines of the PRISMA-ScR checklist for reporting scoping reviews (8), ensuring methodological transparency and rigor. In addition, the methodological framework proposed by the Joanna Briggs Institute (JBI) (9) was adopted, which included the following phases: formulation of the research question, identification of relevant studies, study selection, data extraction, and the synthesis and reporting of results and conclusions. The objective was to map the available evidence on the most prevalent cardiovascular diseases and the risk factors associated with sudden death in amateur athletes.

The research question was structured using the PCC framework: the population included amateur athletes aged 18 years and older (P), the concept encompassed cardiovascular diseases and associated risk factors (C), and the context focused on sudden cardiac death (C). Systematic searches were conducted in the PubMed, SciELO, Scopus, and Springer databases. Study selection was carried out in three phases (title screening, abstract screening, and full-text review with final inclusion) by two independent investigators, who also manually removed duplicate records. Studies were included without restrictions on publication date and in three languages: Spanish, English, and Portuguese. Data were extracted using a standardized table that included author, year, country, study design, population characteristics, identified diseases, risk factors, and relevant events.

### Research question

To conduct this review, the following research question was proposed: *What are the most prevalent cardiovascular diseases and the risk factors that contribute to sudden death in amateur athletes?*

The question was formulated according to the PCC framework as follows:

- P (Population): For the purposes of this scoping review, amateur athletes were defined as individuals who regularly participate in organized or recreational sports without being professional or full-time elite athletes. This definition includes recreational athletes, competitive non-elite athletes, scholastic and collegiate athletes, as well as individuals engaged in organized sports outside professional leagues. Given the absence of a universally accepted definition in the literature, a broad and inclusive approach was intentionally adopted to capture the diversity of non-professional sports participation and to comprehensively map the available evidence.
- C (Concept): Prevalent cardiovascular diseases and associated risk factors
- C (Context): Sudden cardiac death

### Selection criteria

To identify relevant studies, a bibliographic search aligned with the research question was conducted. Studies were selected if they met the following inclusion criteria:

Inclusion criteria

- Primary scientific articles, descriptive or experimental studies, qualitative and quantitative studies, published in Spanish, English, or Portuguese, with no restriction on publication date.

Exclusion criteria

- Studies that did not provide specific information on amateur athletes or that focused exclusively on professional athletes were excluded.
- Studies with unclear methodological designs or that did not directly address the research question were also excluded.

### Information sources

A search for original descriptive articles published in indexed journals and available in full text, without time restrictions, was conducted in the following databases: PubMed, SciELO, Springer, and Scopus. Descriptors from the Health Sciences Descriptors (DeCS) and Medical Subject Headings (MeSH) were used, including: *“Athletes,” “non-professional,” “Sudden Death,” “Cardiac,” “Cardiovascular,” “Diseases,” “Heart,” “Risk Factors,”* and *“Genetics Predispositions,”* combined using the Boolean operators AND and OR.

### Search strategy

The search was conducted using controlled and uncontrolled language, selecting terms based on Medical Subject Headings (MeSH), Health Sciences Descriptors (DeCS), and keywords organized according to the PCC question in Spanish, English, and Portuguese. For each database, specific search equations were constructed, after which two investigators, together with a search specialist, analyzed the retrieved data. To improve data precision, search filters were applied to reduce the number of studies that did not directly address the research question, thereby increasing the exhaustiveness and precision of identifying relevant material. The search equations are presented in Table 1.

**Table 1:** Study search strategy.

Database	Number of records	ST	SR	LC	SF	Search strategy
PUBMED	828	32	7	6	1	(“Sudden Cardiac Death” AND “Risk Factors”) AND (“Athletes”[Mesh] OR sport* OR amateur OR recreational)
SCIELO	181	13	10	3	0	“Sudden cardiac death” AND “Risk factors” OR “Predisposition Genetic”
SPRINGER	770	7	3	2	0	“Cardiovascular Diseases” OR “Heart Diseases” AND “Sudden Cardiac Death” AND “Risk Factors”
SCOPUS	28	13	4	4	4	TITLE-ABS-KEY (“sudden cardiac death” AND (“risk factors” OR “cardiovascular risk”) AND (sport* OR athlete* OR amateur OR recreational))

ST: title screening; SR: abstract screening; LC: full-text review; SF: studies finally included.

To carry out the search, the concepts were standardized in PubMed. Subsequently, an advanced literature search was conducted in the databases listed in Table 1, using DeCS/MeSH terms in English, Spanish, and Portuguese, applying a specific search equation for each database.

### Selection of sources of evidence

The collection of data or bibliographic references was performed using the Mendeley platform by the investigators, with the participation of a third investigator who served as a reviewer, after extracting the articles identified through the search equations applied in each database. The records were downloaded in RIS format and imported into Mendeley. Within the platform, several filters were applied to ensure appropriate article selection. The first filter involved the removal of duplicate records. Subsequently, title and abstract screening was conducted to include articles reporting prevalent cardiovascular diseases and risk factors associated with sudden cardiac death in amateur athletes. Finally, full-text screening was performed, and the studies selected at the final stage had their study variables entered into an Excel matrix (see Table 2) in order to verify compliance with the inclusion criteria.

**Table 2:** Characteristics of the included studies.

No	Year/country/authors	Objective	Study design	Population and sample	Variables assessed	Results	Conclusions	Factors associated with sudden death	Diseases associated with sudden death
1	2009/USA/Maron BJ et al. (10)	To estimate the absolute number of sudden deaths in young athletes in the USA	Retrospective and prospective observational study	1866 athletes aged 8–39 years from 38 sports	Demographics, sport, circumstances, causes of death	56% cardiovascular causes; HCM most frequent	SCD is rare but socially impactful	Intense exercise, male sex, African-American ethnicity	Hypertrophic cardiomyopathy, coronary anomalies
2	2016/Germany/Böhm P et al. (11)	To examine incidence and causes of sports-related SCD	Prospective observational study	144 cases, mainly recreational athletes	Sport type, age, cause of death	Myocarditis (<35y), coronary disease (>35y)	Prevention should target non-elite athletes	Male sex, age >35, intense exercise	Coronary disease, myocarditis
3	2017/Switzerland/Asatryan B et al. (12)	To analyze sport-related and non-sport-related SCD	Retrospective observational study	349 cases aged 10–39 years	Relation to sport, cause, incidence	Coronary artery disease predominant	ECG and risk factor screening recommended	Male sex, intense effort	CAD, cardiomyopathies
4	2014–2018/Worldwide/Egger F et al. (13)	To investigate regional patterns of SCD in football	Prospective observational study	617 football-related SCD cases	Age, cause, survival factors	CAD in >35y; cardiomyopathies in <35y	Need for AED and CPR access	Age, lack of CPR/AED	Coronary disease, cardiomyopathies
5	2021/USA/Peterson DF et al. (14)	To investigate etiology of sudden cardiac arrest in athletes	Prospective surveillance study	331 cases aged 11–29 years	Demographics, etiology, sport	HCM most frequent cause	Improved emergency preparedness needed	Male sex, African-American ethnicity	Hypertrophic cardiomyopathy

### Data extraction

Data extraction from the included studies was performed through detailed reading of the full texts, identifying and organizing relevant information into evidence tables. This process was carried out independently by two investigators, who subsequently compared their results to verify data consistency. In cases of disagreement, a third investigator acted as an arbitrator to review the records and facilitate consensus. For each study, predefined data were extracted according to the variables listed in Table 2.

### Critical appraisal

The methodological quality of the included studies was assessed using the Joanna Briggs Institute (JBI) (9) critical appraisal tool for observational cohort studies, specifically applicable to retrospective cohort designs. This tool is designed to evaluate key domains related to methodological rigor and internal validity, including the clarity of inclusion criteria, validity and reliability of exposure and outcome measurements, identification and management of confounding factors, adequacy of follow-up, and appropriateness of the statistical analysis.

Each study was independently appraised by two reviewers. Disagreements were resolved through discussion, and when necessary, a third reviewer was consulted to reach consensus. For each appraisal item, responses were categorized as “yes,” “no,” “unclear,” or “not applicable,” in accordance with JBI recommendations. Methodological quality scores were calculated as the proportion of criteria fulfilled (“yes” responses) relative to the total number of applicable items, allowing studies to be classified descriptively as having moderate or high methodological quality.

### Presentation of results

**Figure 1:**
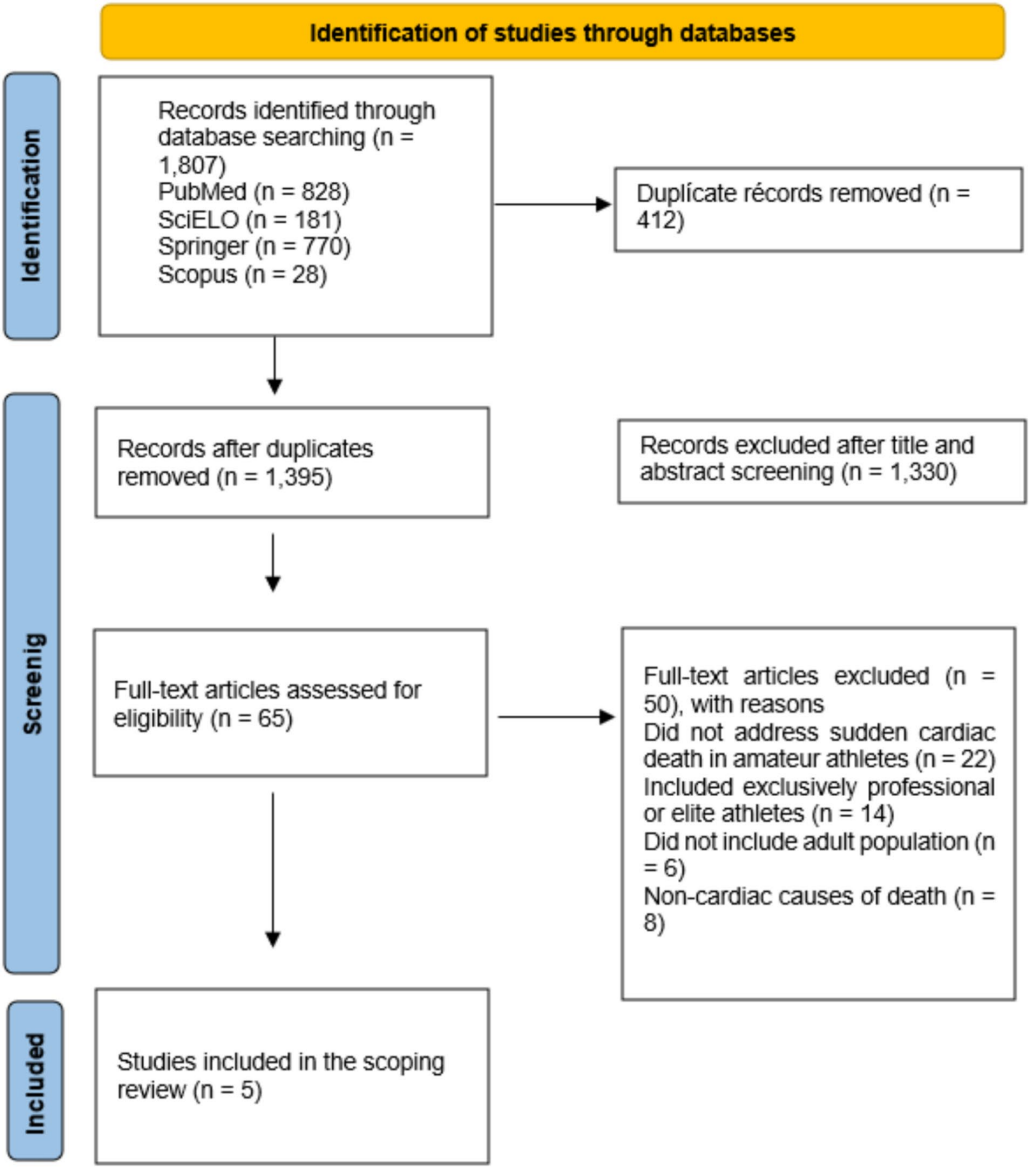
PRISMA-ScR flow diagram of the study selection process.

The results are presented in descriptive tables that include relevant aspects of each study, such as year, country and authors, objective, study design, population and sample, evaluated variables, results, conclusions, factors associated with sudden death, and diseases associated with sudden death. Tables illustrating the search strategy and the search equations used (Table 1), as well as the final number of included studies, are presented. In addition, a flow diagram describing the search process and the final number of included studies is provided (Figure 1).

## Results

A total of 1,807 studies were initially identified. After preliminary screening, titles and abstracts were evaluated, and the inclusion and exclusion criteria were applied. A total of 1,802 studies were excluded, and finally, five articles were included in the review (Figure 1).

All included studies had a descriptive design and were observational cohort studies, both prospective and retrospective (10–14), in which the causes of sudden cardiac death, type of sport or physical activity, and associated risk factors were evaluated. Regarding the origin of the identified studies, two were conducted in the United States (10, 14), one in Germany (11), one in Switzerland (12), and one consisted of an evaluation of autopsy reports from 67 different countries (13).

The most frequently reported cause of death across the studies was hypertrophic cardiomyopathy, accounting for approximately 80% of cases in most studies (10, 11, 13, 14). Other reported causes included arrhythmogenic right ventricular cardiomyopathy, congenital coronary artery anomalies, channelopathies (long QT syndrome and Brugada syndrome), mitral valve prolapse, aortic stenosis, aortic dissection, atherosclerotic coronary artery disease, myocarditis, acute myocardial infarction (10–14), and blunt trauma (commotio cordis) (10).

Regarding sports disciplines, sudden cardiac death was most frequently reported in soccer (10, 13), followed by athletics, basketball, baseball, and American football (10, 13, 14). One study did not specify the sport practiced by the deceased individuals (12). Across all included studies, a marked predominance of male sex was consistently observed, indicating that men represent the group at highest risk for sudden cardiac death in amateur sports settings (10–14).

The mean age at death in study (10) was 18 years, while in studies (11–14) it was 35 years, with variations observed depending on the population and competitive level. One study reported a higher number of deaths in recreational sports (10, 11), whereas another study indicated that the highest incidence of sudden death occurred in individuals who did not practice any type of sport, either recreational or elite (12).

The most frequently reported risk factors for sudden death included a previous diagnosis of cardiovascular disease, smoking, arterial hypertension, dyslipidemia, family history of premature coronary artery disease, coronary artery stenosis, and previous myocardial infarction (10, 12). Additionally, recent respiratory infections associated with myocarditis in young individuals and the absence of early cardiopulmonary resuscitation or defibrillation during events were also identified as relevant risk factors (11–14).

Methodological quality assessment showed that most studies had high methodological quality (11–14), while one study was rated as having moderate methodological quality (10), mainly due to the lack of representative group recruitment and heterogeneous measurement of exposures for the assignment between exposed and non-exposed groups (Table 3).

**Table 3:** Methodological quality assessment of the included studies using the Joanna Briggs Institute (JBI) tool.

Authors	Study design	Quality assessment (JBI tool) (%)	Interpretation
Barry J. Maron, Joseph J. Doerer, Tammy S. Haas, David M. Tierney, Frederick O. Mueller (10)	Retrospective and prospective observational study	Joanna Briggs Institute, 72%	Moderate quality
Philipp Böhm, Jürgen Scharhag, Tim Meyer (11)	Prospective observational study	Joanna Briggs Institute, 82%	High quality
Babken Asatryan, Cristina Vital, Christoph Kellerhals, Argelia Medeiros Domingo, Christoph Gräni, Lukas D. Trachsel, Christian M. Schmied, Ardan M. Saguner, Prisca Eser, David Herzig, Stephan Bolliger, Katarzyna Michaud, Matías Guillermo (12)	Retrospective observational study based on forensic autopsies and official records	Joanna Briggs Institute, 82%	High quality
Egger F, Scharhag J, Kästner A, Dvořák J, Böhm P, Meyer T (13)	Prospective observational study	Joanna Briggs Institute, 85.7%	High quality
Danielle F. Peterson, Kristen Kucera, Leah Cox Thomas, Joseph Maleszewski, David Siebert, Martha Lopez-Anderson, Monica Zigman, Jared Schattenkerk, Kimberly G. Harmon, Jonathan A. Drezner (14)	Prospective surveillance study (4-year follow-up)	Joanna Briggs Institute, 85.7%	High quality

## Discussion

The main objective of this study was to map the available evidence on the most prevalent cardiovascular diseases and the risk factors associated with sudden cardiac death (SCD) in amateur athletes. Hypertrophic cardiomyopathy was identified as the most frequently reported cause, which can be explained by the fact that this condition compromises both the electrical and mechanical function of the heart, favoring the occurrence of fatal ventricular arrhythmias during intense exercise. Similar findings were reported in studies (10, 14), in which hypertrophic cardiomyopathy was identified as the most frequent etiological factor in young athletes.

These findings are consistent with studies conducted in the United States, such as study (10), where the analysis of a large cohort of sudden deaths in young adult athletes confirmed hypertrophic cardiomyopathy as the predominant etiology.

The most frequently reported cause of sudden death in amateur athletes was hypertrophic cardiomyopathy (HCM). This condition is characterized by marked hypertrophy of the interventricular septum and disorganization of myocardial tissue, which alters the normal architecture of muscle fibers. These structural modifications lead to prolonged electrical conduction pathways and increased electrical instability, facilitating the development of malignant ventricular tachyarrhythmias capable of triggering sudden death, particularly during intense physical exertion when myocardial metabolic demand is significantly increased (15).

The pathophysiology of HCM involves multiple mechanisms. On the one hand, myocardial hypertrophy and interstitial fibrosis impair diastolic relaxation and predispose to ischemia; on the other hand, myocyte disarray disrupts electrical conduction, promoting re-entry circuits and fatal ventricular arrhythmias. This scenario is exacerbated during exercise, when catecholamine release and increased myocardial contractility act as critical triggers (16).

Hypertrophic cardiomyopathy is considered predominantly congenital with a genetic basis, associated with mutations in sarcomeric genes such as *MYH7* and *MYBPC3*, which lead to disproportionate hypertrophy of the interventricular septum and myocardial disorganization (17, 18). These structural alterations generate an electrically unstable substrate. Nevertheless, acquired or secondary conditions that may mimic a hypertrophic phenotype have been reported, including hypertrophy induced by chronic arterial hypertension due to sustained ventricular pressure overload, metabolic and storage diseases, or, in specific cases, the physiological hypertrophy observed in the “athlete’s heart.” Overall, HCM combines mechanical alterations (outflow tract obstruction and diastolic dysfunction) and electrical disturbances (re-entry circuits and arrhythmias) that increase the risk of sudden death (2, 19).

Hypertrophic cardiomyopathy represents a particular risk because it may remain asymptomatic for years, without evident clinical signs, until intense exertion triggers the first event, which is often sudden cardiac death. The danger lies in the fact that individuals considered “healthy” may begin sports practice without knowing they have this condition, thereby being exposed to malignant ventricular arrhythmias or circulatory collapse during exercise. For this reason, several scientific societies recommend the implementation of preventive cardiovascular screening, including tests such as electrocardiography and echocardiography, before engaging in high-intensity physical activities (20). These screening strategies allow the detection of structural or electrical cardiac abnormalities that, if diagnosed early, could significantly reduce sudden cardiac deaths in both competitive and recreational sports settings.

Other relevant causes of sudden death identified in the studies were atherosclerotic coronary artery disease, more frequent in athletes older than 35 years, and myocarditis, particularly observed in younger individuals with recent respiratory infections. Both conditions create a substrate for fatal events, either through myocardial ischemia, acute myocardial infarction, or secondary ventricular arrhythmias. These findings are consistent with international reports, such as the prospective German study, which described coronary artery disease as the leading cause in middle-aged adult athletes, whereas myocarditis predominated in those younger than 35 years (11). Similarly, studies conducted in Switzerland confirmed premature coronary artery disease as the most frequent etiology of sudden death in young populations, reinforcing the importance of integrating screening for classical cardiovascular risk factors and early detection of viral infections before returning to competitive sports (12).

The sports discipline with the highest number of reported deaths was soccer. This sport imposes very high cardiovascular demands, as players often perform at intensities close to 90% of their maximum heart rate while sustaining prolonged periods of exertion. This increases the risk of triggering events such as ventricular arrhythmias, myocardial ischemia, or infarction, especially in the presence of underlying cardiac conditions. These findings are consistent with those reported by Işın et al. (2021) in Turkey, where 82% of soccer-related sudden deaths occurred during recreational practice, with considerably higher attributable mortality in men aged 30 to 49 years (21).

The mean age at death was 29 years, indicating that SCD does not only affect older adults but also young individuals in their peak athletic years. This may be explained by the interaction between genetic predisposition and exposure to high-intensity physical exertion. Age was also reported as a determining factor, with differences in etiology between individuals younger and older than 35 years (11, 14).

Across the analyzed studies, the most frequently reported risk factors for sudden death were previous diagnoses of cardiovascular disease, such as coronary artery stenosis or prior myocardial infarction, as well as classical predisposing conditions including smoking, arterial hypertension, dyslipidemia, and family history of premature coronary artery disease. These findings reinforce that, even in physically active individuals, traditional cardiovascular risk factors continue to play a central role in the development of fatal events. In younger individuals, recent respiratory infections were consistently associated with myocarditis, demonstrating how seemingly benign inflammatory processes can become critical triggers during intense physical exertion (22). Furthermore, the absence of cardiopulmonary resuscitation or early defibrillation at the event site was associated with increased mortality, highlighting the importance of secondary prevention strategies. In addition, non-modifiable factors such as male sex, African American ethnicity, and participation in high-risk sports such as basketball or American football at high competitive levels were more frequently associated with sudden death. These findings align with international literature and suggest that the interaction between genetic predisposition, cardiovascular comorbidities, and sport-specific demands creates a high-risk scenario requiring targeted preventive measures in the amateur population (23).

This study provides relevant evidence by highlighting the need for cardiovascular screening in amateur athletes, including electrocardiography, echocardiography, and family history assessment, as well as educational programs in cardiopulmonary resuscitation and the availability of automated external defibrillators (AEDs) in sports settings (10, 12, 13, 23).

Overall methodological quality of the included studies ranged from high to moderate, indicating that the results are consistent and reliable, although heterogeneity in data collection methods and differences between countries should be acknowledged (10–14).

The strengths of this study lie in the integration of evidence from different regions, including Germany, the United States, and 67 countries, as well as multiple sports such as soccer, athletics, basketball, and baseball, providing a global perspective on the problem and highlighting common factors that may guide preventive strategies. In addition, structured information search protocols and methodological quality assessment of the included studies were applied (10–14).

The limitations of this study include the small number of selected studies, potential underreporting of cases in official registries, and lack of uniformity in diagnostic and autopsy criteria across countries, limitations that have also been reported in previous reviews of sports-related sudden cardiac death (24).

Future research should expand scientific production in non-professional sports contexts, where the risk of sudden death may be underestimated and preventive measures are less stringent than in elite sports. In addition, it is necessary to explore the influence of factors such as training level, absence of systematic cardiovascular screening, and the coexistence of comorbidities common in the general population, in order to develop prevention strategies tailored to this group of athletes.

## Conclusion

Hypertrophic cardiomyopathy was identified as the main cause of sudden cardiac death in amateur athletes, followed by atherosclerotic coronary artery disease and myocarditis.

The main risk factors attributed to sudden death were arterial hypertension, dyslipidemia, previous myocardial infarction, coronary artery stenosis, and recent respiratory infections, as well as non-modifiable factors such as family history and participation in high-intensity sports, including the absence of automated external defibrillators (AEDs) in sports practice settings in the event of a sudden cardiac event.

## Data Availability

The raw data supporting the conclusions of this article will be made available by the authors, without undue reservation.

## References

[1] Egger F Ukaj A Hollander K. Sudden cardiac death in sports. Dtsch Z Sportmed. (2023) 74:14–8. doi: 10.5960/dzsm.2022.550

[2] Finocchiaro G Westaby J Sheppard MN Papadakis M Sharma S. Sudden cardiac death in young athletes. J Am Coll Cardiol. (2024) 83:350–70. doi: 10.1016/j.jacc.2023.10.032, 38199713

[3] Villar-Gómez FM Gómez-Salgado J Fernández-García D Dias A García-Iglesias JJ Ruiz-Frutos C. Prevención de la muerte súbita cardiaca en el deportista joven desde la perspectiva enfermera. RqR Enfermería Comunitaria (Revista de SEAPA). (2020) 8:36–45.

[4] Bonilla JC Parra-Medina R Polo JF Rocha JE Téllez JP Chaves JJ . Análisis clínico e histopatológico de la prevalencia de enfermedades cardiacas en muerte súbita. Estudio en autopsias. Repert Med Cir. (2022) 31:161–9. doi: 10.31260/RepertMedCir.01217372.1244

[5] Alfaro Díaz M Eisen Jofré D Antezana Bilbao La Vieja G Bustamante S. Fístulas Coronarias Múltiples. Revisión del tema, a propósito de 2 casos. RETIC. (2022) 5:5–10. doi: 10.37615/retic.v5n3a2

[6] McHugh C Hind K Cunningham J Davey D Wilson F. A career in sport does not eliminate risk of cardiovascular disease: a systematic review and meta-analysis of the cardiovascular health of field-based athletes. J Sci Med Sport. (2020) 23:792–9. doi: 10.1016/j.jsams.2020.02.009., 32139313

[7] Miguel Gonçalves C Vazão A Carvalho M Cabral M Martins A Martins H . Sudden cardiac death in athletes: a 20-year analysis in Portugal. Rev Port Cardiol. (2024) 44:77–83. 39393636 10.1016/j.repc.2024.08.010

[8] Page MJ McKenzie JE Bossuyt PM Boutron I Hoffmann TC Mulrow CD . Declaración PRISMA 2020: una guía actualizada para la publicación de revisiones sistemáticas. Rev Esp Cardiol. (2021) 74:790–9. doi: 10.1016/j.recesp.2021.06.016 34446261

[9] Institute TJB. Joanna Briggs institute reviewers’ manual: 2014 edition. Adelaide: Joanna Briggs Institute (2014).

[10] Maron BJ Doerer JJ Haas TS Tierney DM Mueller FO. Sudden deaths in young competitive athletes: analysis of 1866 deaths in the United States, 1980-2006: analysis of 1866 deaths in the United States, 1980-2006. Circulation. (2009) 119:1085–92. doi: 10.1161/CIRCULATIONAHA.108.804617, 19221222

[11] Bohm P Scharhag J Meyer T. Data from a nationwide registry on sports-related sudden cardiac deaths in Germany. Eur J Prev Cardiol. (2016) 23:649–56. doi: 10.1177/2047487315594087, 26130495 PMC4776219

[12] Asatryan B Vital C Kellerhals C Medeiros-Domingo A Gräni C Trachsel LD . Sports-related sudden cardiac deaths in the young population of Switzerland. PLoS One. (2017) 12:e0174434. doi: 10.1371/journal.pone.0174434, 28350812 PMC5370100

[13] Egger F Scharhag J Kästner A Dvořák J Bohm P Meyer T. FIFA sudden death registry (FIFA-SDR): a prospective, observational study of sudden death in worldwide football from 2014 to 2018. Br J Sports Med. (2022) 56:80–7. doi: 10.1136/bjsports-2020-102368, 33361135

[14] Peterson DF Kucera K Thomas LC Maleszewski J Siebert D Lopez-Anderson M . Aetiology and incidence of sudden cardiac arrest and death in young competitive athletes in the USA: a 4-year prospective study. Br J Sports Med. (2021) 55:1196–203. doi: 10.1136/bjsports-2020-102666, 33184114 PMC8551972

[15] Badura K Buławska D Dąbek B Witkowska A Lisińska W Radzioch E . Primary electrical heart disease-principles of pathophysiology and genetics. Int J Mol Sci. (2024) 25:1826. doi: 10.3390/ijms25031826, 38339103 PMC10855675

[16] Shah AB Bechis MZ Brown M Finch JM Loomer G Groezinger E . Catecholamine response to exercise in patients with non-obstructive hypertrophic cardiomyopathy. J Physiol. (2019) 597:1337–46. doi: 10.1113/JP277494, 30552684 PMC6395414

[17] Hong Y Xi HT Yang XY Su WW Li XP. Pathogenic genes and clinical prognosis in hypertrophic cardiomyopathy. World J Cardiol 2025; 17:99595. doi: 10.4330/wjc.v17.i1.99595 39866219 PMC11755131

[18] Wasfy MM Hutter AM Weiner RB. Sudden cardiac death in athletes. Methodist Debakey Cardiovasc J. (2016) 12:76–80. doi: 10.14797/mdcj-12-2-76, 27486488 PMC4969030

[19] D’Ambrosio P De Paepe J Janssens K Mitchell AM Rowe SJ Spencer LW . Arrhythmias and structural remodeling in lifelong and retired master endurance athletes. J Sport Health Sci. (2025) 14:101043. doi: 10.1016/j.jshs.2025.101043, 40273982 PMC12305178

[20] Oxborough D George K Cooper R Bhatia R Ramcharan T Zaidi A . Echocardiography in the cardiac assessment of young athletes: a 2025 guideline from the British Society of Echocardiography (endorsed by cardiac risk in the young). Echo Res Pract. (2025) 12:7. doi: 10.1186/s44156-025-00069-0, 40083035 PMC11907977

[21] Işın A Güvenç O Güvenç S. Epidemiology of football-related sudden cardiac death in Turkey. Medicina. (2021) 57:1105. doi: 10.3390/medicina57101105, 34684142 PMC8540717

[22] Van Name J Wu K Xi L. Myocarditis - a silent killer in athletes: comparative analysis on the evidence before and after COVID-19 pandemic. Sports Med Health Sci. (2024) 6:232–9. doi: 10.1016/j.smhs.2024.03.003, 39234482 PMC11369839

[23] Vora A Burkule N Contractor A Bhargava K. Prevention of sudden cardiac death in athletes, sportspersons and marathoners in India. Indian Heart J. (2018) 70:137–45. doi: 10.1016/j.ihj.2017.12.004, 29455769 PMC5903013

[24] Han J Lalario A Merro E Sinagra G Sharma S Papadakis M . Sudden cardiac death in athletes: facts and fallacies. J Cardiovasc Dev Dis. (2023) 10:68. doi: 10.3390/jcdd10020068, 36826564 PMC9965876

[25] Priori SG Blomström-Lundqvist C Mazzanti A Blom N Borggrefe M Camm J . 2015 ESC Guidelines for the management of patients with ventricular arrhythmias and the prevention of sudden cardiac death. Eur Heart J. (2015) 36:2793–867. doi: 10.1093/eurheartj/ehv316 26745817

